# Innovative high fat diet establishes a novel zebrafish model for the study of visceral obesity

**DOI:** 10.1038/s41598-024-53695-9

**Published:** 2024-02-06

**Authors:** Katarzyna Smolińska, Jan Sobczyński, Aleksandra Szopa, Artur Wnorowski, Ewa Tomaszewska, Siemowit Muszyński, Anna Winiarska-Mieczan, Tomasz Czernecki, Agata Bielak, Katarzyna Dobrowolska, Kacper Smoliński, Renata Klebaniuk, Piotr Dobrowolski

**Affiliations:** 1 Chronic Wounds Laboratory, Medical University of Lublin, Chodźki St. 7, 20-093 Lublin, Poland; 2 Department of Clinical Pharmacy and Pharmaceutical Care, Medical University of Lublin, Chodźki St. 1, 20-093 Lublin, Poland; 3 Department of Biopharmacy, Medical University of Lublin, 4A Chodźki St. 4A, 20-093 Lublin, Poland; 4 Department of Animal Physiology, Faculty of Veterinary Medicine, University of Life Sciences in Lublin, Akademicka St. 12, 20-950 Lublin, Poland; 5 Department of Biophysics, University of Life Sciences in Lublin, Akademicka St. 13, 20-950 Lublin, Poland; 6 Institute of Animal Nutrition and Bromatology, University of Life Sciences in Lublin, Akademicka St. 13, 20-950 Lublin, Poland; 7 Department of Biotechnology, Microbiology and Human Nutrition, Dietitian Service, Faculty of Food Science and Biotechnology, University of Life Sciences in Lublin, Skromna 8, 20-704 Lublin, Poland; 8 Faculty of Biology and Biotechnology, Maria Curie Sklodowska University, Akademicka St. 19, 20-033 Lublin, Poland; 9 Faculty of Biology, Warsaw University, Żwirki I Wigury St. 101, 02-089 Warsaw, Poland; 10 Department of Functional Anatomy and Cytobiology, Maria Curie Sklodowska University, Akademicka St. 19, 20-033 Lublin, Poland

**Keywords:** Developmental biology, Animal disease models

## Abstract

Obesity is a complex chronic condition associated with multiple health risks, including visceral obesity, which is particularly detrimental. To gain insight into the mechanisms underlying obesity and its associated pathologies, a novel zebrafish model was established using an innovative high-fat diet (HFD). The primary goal was to induce visceral obesity in zebrafish and study the associated structural changes. To achieve this, a unique HFD consisting of 40% beef fat (HFD40) was developed and supplemented with magnesium aluminometasilicate to improve stability in a high humidity environment. Feeding regimens were initiated for both juvenile (starting at 2 weeks post-fertilization, lasting 18 weeks) and adult zebrafish (3 months post-fertilization, 8 weeks feeding duration). The innovative dietary approach successfully induced visceral obesity in both juvenile and adult zebrafish. This new model provides a valuable tool to study obesity-related pathologies, metabolic syndrome, and potential therapeutic interventions. Most importantly, the low-cost and easy-to-prepare composition of HFD40 was seamlessly incorporated into the water without the need for separation, was readily absorbed by the fish and induced rapid weight gain in the zebrafish population. In conclusion, this study presents a novel HFD40 composition enriched with a high beef fat concentration (40%), which represents a significant advance in the development of an experimental zebrafish model for the study of visceral obesity and associated metabolic changes.

## Introduction

A balanced diet is a key indicator of a healthy lifestyle. The Mediterranean Diet, which is renowned for its rich seafood and fish content, is considered among the best^1^. Over the years, numerous clinical studies have demonstrated that this dietary approach provides sustained health benefits for individuals suffering from cardiovascular conditions, type 2 diabetes, metabolic syndrome, obesity, and cancer^1–4^. Fish and seafood contain a significant proportion of Omega-3 fatty acids (namely eicosapentaenoic acid -EPA- and docosahexaenoic acid –DHA). When incorporated to an adequate level, EPA and DHA affect the physical properties of cell membranes and membrane protein-mediated responses, cell signaling and gene expression in various cell types^5^. EPA and DHA exert their effects on cell and tissue physiology, and on how cells and tissues respond to external signals. In most instances, improvements in disease biomarker profiles or health-related outcomes are compatible with observable effects^5^. Additionally, noteworthy indicators include the proportion of proteins, fat, and carbohydrates in the diet^6^. On the contrary, the so-called "Western diet" which has a high proportion of carbohydrates and fat is a contributing factor to two major epidemics: cancer and obesity. The consumption of "junk food", which is high in sugar and fat, is a major cause^7–11^.

One of the most pressing problems facing modern societies worldwide is obesity, a global health risk that affects more than 1 billion people. This rising crisis affects both adults and children^12^. This chronic condition, characterized by excessive accumulation of adipose tissue, has far-reaching implications as adipose tissue acts as an active endocrine organ, influencing multiple metabolic pathways^13,14^. Notably, obesity is strongly associated with serious cardiovascular and metabolic complications, particularly visceral obesity, which significantly increases the risk of insulin resistance and diabetes^15^. Visceral obesity is the accumulation of fat in the abdominal cavity and is closely associated with several health problems, including inflammation of the intestines^16^.

To gain deeper insights into the underlying mechanisms of visceral obesity and related pathologies, in particular intestinal inflammation^16^, an appropriate animal model is essential. Zebrafish, with their high degree of conservation in the distribution and formation of adipose tissue compared to mammals, are suitable for studying obesity in both larval and adult stages, making them a proper animal model for obesity research^17^. By administering a high-fat diet (HFD), researchers can induce a zebrafish model of obesity, facilitating the study of obesity pathogenesis and the screening of potential anti-obesity drug candidates^18^.

In zebrafish obesity models, researchers commonly use dry egg yolk or different types of oils and *Artemia salina* as the primary fat sources^17^. Dry egg yolk, with a fat concentration of around 60% (Sigma Aldrich), and *Artemia salina*, a vital protein source^19^, have been instrumental in inducing hyperlipidaemia and increased body weight in fish^20^. In particular, high levels of *Artemia salina* in the diet have been associated with the development of hypertriglyceridemia and hepatosteatosis^21^. However, it is worth noting that none of the zebrafish experiments using these diets have successfully reproduced the typical features of visceral obesity observed in humans. This limitation is a critical drawback of existing zebrafish obesity models^21^.

Recognizing these limitations, we hypothesized that introducing a diet enriched in animal fat into the zebrafish diet could promote visceral adipose tissue growth regardless of the developmental stage of the fish. To test this hypothesis, we conducted experiment with juvenile and adult zebrafish, introducing diets enriched with 40% and 60% beef fat. Our study aimed to determine which diet composition could effectively induce typical visceral obesity after 18 and 8 weeks of feeding, and whether the newly formulated animal fat-enriched diet could be effectively implemented under aquatic conditions.

## Materials and methods

### Ethics

The study was approved by the Local Ethics Committee for Animal Experiments (University of Life Sciences in Lublin, Poland, No. 100/2019). The methods were carried out in accordance with norms of the European Union law (Directive 2010/63/UE on the protection of animals used for scientific purposes, received in Poland by Legislative Decree 266/2015). The experiments were carried out at the Experimental Medicine Center of the Medical University of Lublin, Lublin, Poland in compliance with the ARRIVE guidelines.

### Zebrafish husbandry

Wild type zebrafish were used in this study. All fish were maintained under standard laboratory conditions at 28 °C with a 14-h light/10-h dark cycle^19^.

### Feed

A commercially available diet, GEMMA (SKRETTING, Stavanger, Norway), consisting of pellets of different sizes (75 µm, 150 µm, and 300 µm) tailored to the specific developmental stages of zebrafish, was used to formulate both the control diet and the experimental HFD. The experimental HFD, characterized by a modified composition (patent number Pat. 242859), was designed to contain an additional 40% or 60% fat content. This was achieved by incorporating beef fat in powdered form Biff Fat Powder (GRAUGmbH, Isselburg, Germany). The beef fat powder used in this diet had a composition of 80% fat, 2% ash, 3% protein and 14% glucose. Importantly, beef fat powder is commercially used as a dietary supplement for animals with low appetite and malnutrition. To improve the stability of the HFD in a high moisture environment, magnesium aluminometasilicate (Neusilin^®^ UFL 2, Fuji Chemical Industries, Tokyo, Japan) was introduced into the formulation. Commonly used in the chemical and pharmaceutical industries, Neusilin^®^ UFL 2 is a porous material known for its high specific surface area (300 m2/g). This unique property allows for the absorption of approximately 3.2 ml/g of oils and fats^22^. This diet composition served as a critical element in the study to investigate the effects of high fat diets on zebrafish and their potential to induce visceral obesity, regardless of the developmental stage of the fish. The differential comparison of control and experimental diets are presented in Table 1.

**Table 1:** Components of normal (NOD)^#^ and high-fat diet (HFD40, HFD60).

Components	NOD	HFD40	HFD60
Proteins [%]	59	29.5	17.7
Oil [%]	14	7.0	4.2
Ash [%]	14	7.0	4.2
Beef fat [%]	0	40	60
Fibre [%]	0.20	0.10	0.08
Phosphorus [%]	1.30	0.65	0.39
Magnesium aluminometasilicate [%]	0	10	10

^#^Composition of GEMMA (SKRETTING, Stavanger, Norway): calcium 1.5%; sodium 0.7%; vitamin A 23,000 UI/kg; vitamin D3 2800 UI/kg; vitamin C 1000 mg/kg; vitamin E 400 mg/kg.

### Study design

**Figure 1:**
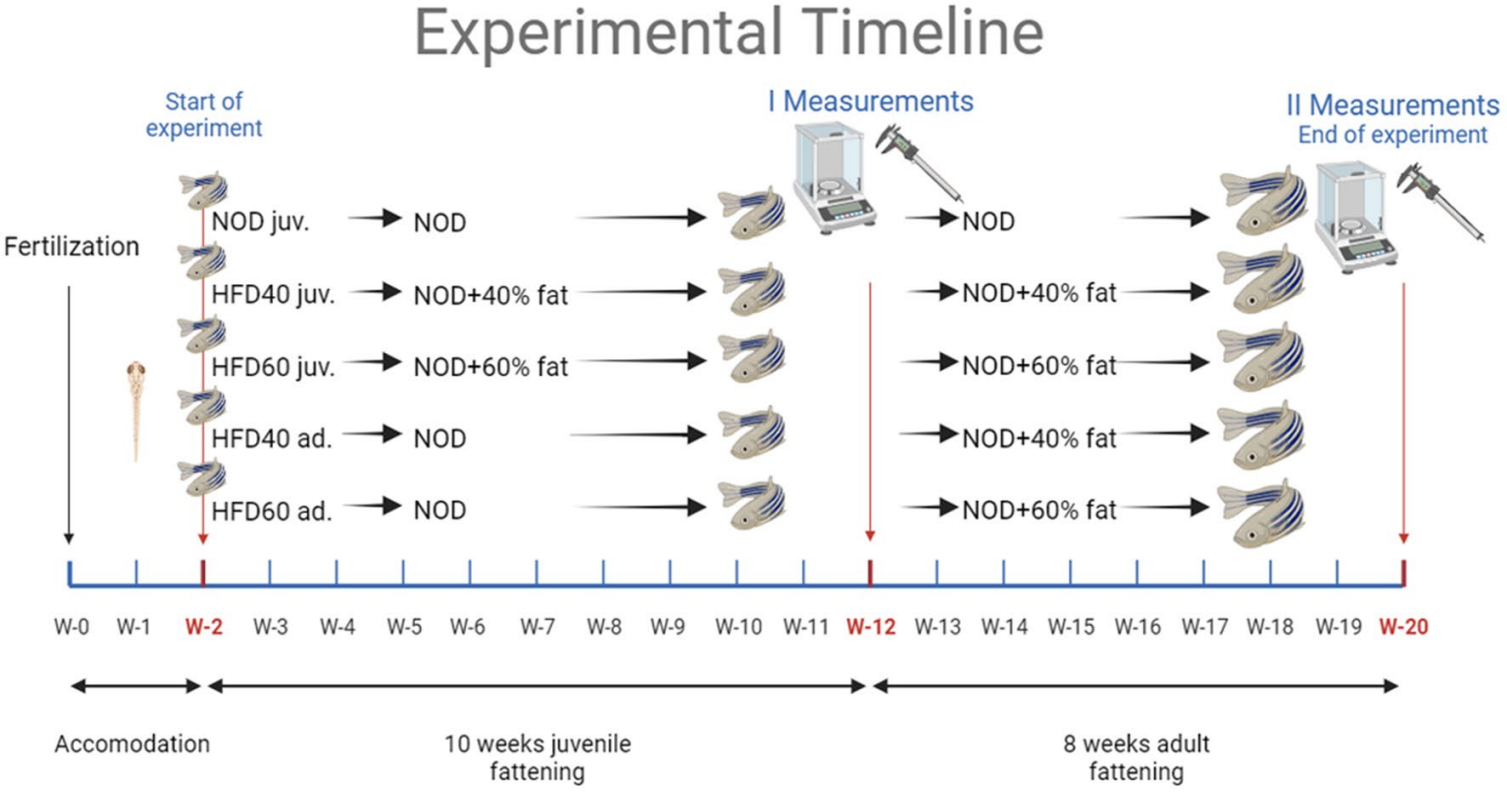
Overview of zebrafish feeding trial and dietary groups. This figure illustrates the experimental setup of the zebrafish feeding trial, assessing the nutritional impact of various diets. All subjects were provided with five daily feedings of their specific diets, each supplemented with a daily serving of *Artemia salina*. The study included: NOD Control Group: Maintained on a GEMMA standard diet (SKRETTING, Stavanger, Norway) with 14% fat. HFD40 Juvenile (HFD40 juv.) and HFD60 Juvenile (HFD60 juv.) Groups: Started high-fat diets from the second week post-fertilization and fed diets with 40% and 60% Beef Fat Powder (GRAU GmbH, Isselburg, Germany), each blended with 10% magnesium aluminometasilicate. HFD40 Adult (HFD40 ad.) and HFD60 Adult (HFD60 ad.) Groups: Began high-fat diets from the 12th week post-fertilization, fed identical compositions as their juvenile counterparts, with corresponding 40% and 60% Beef Fat Powder concentrations. Each group's dietary regimen reflects a distinct blend tailored to evaluate the effects of varying fat concentrations on zebrafish development and health. Figure generated using BioRender.com.

Two-week-old zebrafish larvae were randomly assigned to three dietary groups of 30 fish each, at a stage where sex differentiation was not yet feasible. The control group (NOD) received a standard commercial diet GEMMA with a fat concentration of 14% and supplemented with *Artemia salina*. In parallel, the HFD40 group was fed a newly formulated diet consisting of the commercial basal diet blended with 40% Biff Fat Powder and 10% magnesium aluminometasilicate with simultaneous provision of *Artemia salina*. Similarly, the HFD60 group received a freshly prepared diet consisting of the commercial diet, 60% Biff Fat Powder, 10% magnesium aluminometasilicate and simultaneous provision of *Artemia salina*. All diets were offered ad libitum through mechanical feeders—Eheim AutoFeeder (Deizisau, Germany). Zebrafish received five daily feedings ad libitum, with *Artemia salina* administered once daily. Subsequently, two groups of zebrafish were given diet consisting of a commercial basal diet with simultaneous provision of *Artemia salina* until three months post-fertilization and then switched into diet with animal fat (either 40% or 60%), and magnesium aluminometasilicate at a concentration of 10%, along with *Artemia salina*, starting at three months post-fertilization. These zebrafish were also fed five times daily, ad libitum, using mechanical feeders, with *Artemia salina* supplementation once daily. At the end of either 12 or 20 weeks of life, zebrafish were fasted overnight before being measured and weighed, and finally euthanized at 20 weeks of life by immersion in Tricaine solution at a concentration of 60 µg/ml for 1 min. Intestinal samples were collected from 15 individuals from each group and fixed for subsequent analyses, and the remaining 15 individuals from each group were whole body fixed for visceral fat content measurements. Buffered formalin (pH 0.7) was used as the fixative in both cases. The study design and timeframe of the experiment are shown in Fig. 1.

### Physical analysis and preparation of HFD

The preparation and storage of HFD40 and HFD60 are described in patent number Pat. 242859. The physical analyses carried out to verify the tapped density, the sedimentation time measurement and the effect of particle size are also described in detail in patent number Pat.242859. Briefly, the feed samples, representing each feed composition, underwent sieve analysis using the AS200 sieve tester (Retsch, Germany) and were tested for tapped density using the SVM 222 Tapped Density Tester (Erweka, Germany). Both tests were conducted in accordance with the recommended guidelines outlined in the 11th edition of the European Pharmacopoeia (Ph. Eur.). Sedimentation time measurements were analyzed by measuring the sedimentation time in a 250 ml beaker containing 100 ml of distilled water at a temperature of 25 °C. The MS-H280-Pro magnetic stirrer (Chemland, Poland) was used. The sedimentation time of each sample was recorded, and the average of five measurements was calculated.

### Chemical analyses of commercial diet, HFD40 and HFD60

The basic chemical composition (raw protein, raw ash, raw fat) of basal diet, beef fat, HFD40 and HFD60was analyzed using the AOAC method (AOAC 2000) crude protein by Kjeldahl’s method, crude fat by Soxhlet extraction, crude ash using the mineralization method in a muffle furnace (550 °C)^23^.The content of fatty acids in the feed was determined by gas chromatography in a Varian CP-3800GC-FID apparatus (Varian, Middelburg, The Netherlands)(characteristic of the capillary column: type CP WAX52CB, DF 0.25 mm 60 m, flow rate of helium carrier − 1.4 mL/min, column temperature 120 °C gradually increasing by 2 °C/min up to 210 °C, determination time 120 min., detector FID temperature 260 °C, other gases—hydrogen and oxygen),using the standards Supelco 37 FAME Mix 47,885-U (Sigma-Aldrich, Gillingham, UK), following previous extraction of fat by Soxhlet’s method in a Velp SER 148 apparatus (Velp, Usmate, Italy).

### Analysis of amino acids in feed

Acid hydrolysis of proteins to determine amino acid composition without oxidation was performed according to M.G. Davis and A.J. Thomas^24^.

The ground samples were hydrolyzed at 110 °C for 20 h in 6N HCl using an Ingos HB 016 hydrolyser (INGOS s.r.o., Prague, Czech Republic). After cooling, the samples solutions were filtered through a G-5 funnel (Chemland, Standard, Poland). The hydrolysates were then evaporated on an RVO 400 vacuum evaporator (INGOS s.r.o., Prague, Czech Republic). The dry residue from the vacuum flask was dissolved in citrate buffer, pH 2.2. The prepared samples, after filtering through a syringe filter, were dispensed onto an Ingos AAA 400 amino acid analyzer column (INGOS s.r.o., Prague, Czech Republic).

Protein hydrolysis for the separation of sulfur amino acids was performed according to F. Schramm and S. Moor^25^. Cysteine was oxidized to cysteic acid and methionine to methionine sulphone using peracetic acid, with which the sample was flooded and left overnight in a freezer. The next day, the mixture was flooded with 40% HBr. It was then concentrated on an RVO 400 vacuum evaporator, then flooded with 6N HCl and transferred to an HB 016 Ingos hydrolyser thimble. Hydrolysis was carried out as above.

For the determination of tryptophan, the sample was subjected to alkaline hydrolysis. This is the method according to Slawinski and Tyczkowska^26^. The ground sample is hydrolyzed in a solution of Ba(OH)_2_ using an HB 016 Ingos hydrolyser. The sample is then acidified with 6N HCl and a solution of Na_2_SO_4_ is added to precipitate barium ions. The contents were transferred to phalcones and centrifuged to precipitate BaSO_4_ over 15 min at 3000 × *g* using a ROTOFIX 32A centrifuge (Hettich, Beverly, Massachusetts, USA). The supernatant, after filtration through a syringe filter, was dispensed onto an AAA 400 amino acid analyzer (INGOS s.r.o., Prague, Czech Republic).

Amino acids were determined using an amino acid analyzer AAA 400 (Ingos, Czech Republic, Prague). Amino acids were separated by ion exchange chromatography. A 0.37 × 45 cm column was filled with a resin ion exchanger. Ostion LG ANB ion exchanger (INGOS s.r.o., Prague, Czech Republic) was used for the hydrolysates. It is a strong cation exchanger with an average grain size of approximately 12 µm in the form of Na. Column temperatures of 60 °C and 74 °C. The apparatus detected amino acids by upregulation with ninhydrin (detection reagent). Identification of amino acids was carried out by photometric detection at 570 nm for all amino acids, 440 nm for proline only. Four buffers were used for separation: 1-pH 2.6; 2-pH 3.0; 3-pH 4.25; 4-pH 7.9. After separation of the amino acids, the column was regenerated with 0.2N NaOH.

### Body measurements and BMI calculation

The body weight and length of adult zebrafish was measured first time three months post-fertilization (fish were 12 weeks old) and second time at the end of the experiment (fish were 20 weeks old- Fig. 1). Briefly, the fish were anesthetized with buffered Tricaine^19^. Tricaine was prepared at 4 µg/ml^27^ concentration in facility water and the fish were transferred shortly before taking the amusements. Body weight (g) was measured after the body surface was dried with soft tissue paper using OHAUS Pioneer PX125D weight machine with an accuracy of 0.0001 g. Body length (mm) was measured from the head to the end of the body of zebrafish using digital caliper 1300EAPB (FACOM, Warsaw, Poland) with an accuracy of 0.01 mm. Fulton's Condition Factor (K)^28^ widely used metric in fisheries biology was also calculated as:

$$K = \left( {W/L^{3} } \right) \, \times 100,$$

where: K = Fulton's condition factor, W = Weight of the fish (in grams), L = Length of the fish (in centimeters).

Fulton's condition factor provides information about the relative plumpness or thinness of a fish for its length. A value of 1.0 represents an "average" condition. BMI was calculated using the formula (g)/(cm)^2^^28^. After the first measurement, fish were allowed to recover from the anesthesia. Following the second measurement, fish were sacrificed using buffered tricaine at a concentration of 60 µg/ml^27^. Tissue samples were subsequently taken for further testing (see Fig. 1).

### Visceral fat measurement

**Figure 2:**
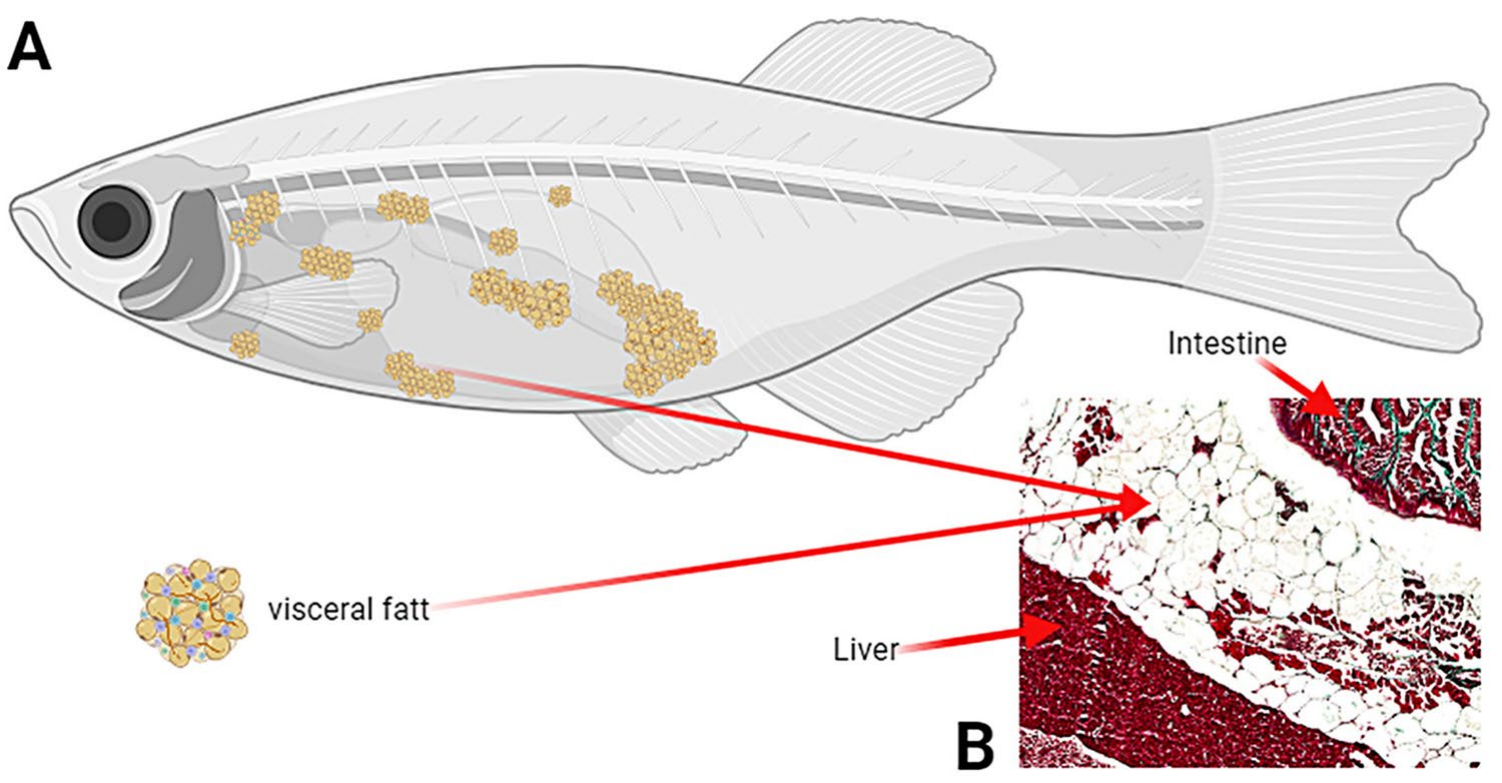
(**A**) Schematic spatial distribution of visceral fat. (**B**) Random example of histological images from the current visceral fat analysis, Goldner staining, magnification × 100. Figure 2. A created using BioRender.com.

Whole sagittal sections of formalin-fixed, paraffin-embedded zebrafish, 5 µm thick, were prepared on a histological microtome (HM360, Microm, Walldorf, Germany). Five sections at 15 µm intervals were cut from each specimen, mounted on a glass polylisine-coated slide, and stained with Goldner's trichrome stain to reveal differences between tissues and organs in the zebrafish abdominal cavity^29,30^. High-resolution scans were taken of each section, covering the entire abdominal cavity, using a BX63 microscope (Olympus, Tokyo, Japan) and an × 10 objective. Images were acquired using the graphical analysis software CellSens Olympus version 1.5 (OLYMPUS, Tokyo, Japan). Machine learning based differential analysis of adipose tissue from the zebrafish abdominal cavity area (Fig. 2) was performed on each scan image using the graphical analysis software Fiji—ImageJ 1.54f. (National Institutes of Health, Bethesda, MD, USA; available at: http://rsb.info.nih.gov/ij/index.html) as previously described^31,32^. Trainable Weka Segmentation (TWS), a plugin available within Fiji (ImageJ), was utilized for this analysis. This tool was implemented to categorize images into classes and the first step involved manually annotating and selecting classes on the learning images. Four distinct tissue and background classes were defined in this study: adipose tissue, other tissues, organs, and background. Manual annotations were added for each class within the TWS graphical user interface for the zebrafish abdominal cavity image. Following this, the classifier training step was executed using the "Train classifier" function in TWS, which initiated a learning process to segment the image into the preselected classes based on the provided annotations. During the refinement and validation phase, annotations for each class were iteratively reviewed and any misclassifications were corrected. The classifier went through multiple training iterations and was validated by an experienced histopathologist to guarantee precise segmentation. Subsequently, the quantification of adipose tissue was carried out by generating a final image with distinct colors assigned to classified pixels of each class. To determine the proportion of adipose tissue, the pixel count of adipose tissue was divided by the total pixel count of adipose tissue, other tissues, organs, and background. After segmenting one reference image, the classifier and its data were saved and imported for analysis of subsequent images. This approach enabled exact and dependable measurement of adipose tissue in images of zebrafish abdominal cavities, verified by skilled histopathologists during training of the classifier^31,32^.

Adipocyte analysis was performed utilizing ImageJ software. The process involved selecting regions of interest (ROI) containing adipocytes on histological images. Following ROI selection, a custom script facilitated the segmentation and quantification of adipocytes. The functions of the script included enhancing contrast, background subtraction, despeckling, thresholding, and particle analysis, followed by the skeletonization function and Summarize Skeleton tool, which collectively enabled the counting of adipocyte numbers and measurement of membrane lengths. The adipocytes were identified based on size and circularity parameters to ensure accuracy.

To validate the semi-automated quantification obtained from the script, we manually counted adipocytes using the 'Cell counter' tool in Fiji—ImageJ. Two independent observers (histologists) who were blinded to the image code performed counts. This tool allowed for meticulous confirmation of adipocyte numbers by direct user interaction with the images, serving as a reliable cross-check against automated counts. This dual approach, combining automated and manual analyses, enhanced the robustness of our adipocyte quantification and provided a thorough validation of our morphometric data.

### Histological assessment

Liver histological assessments were conducted to evaluate hepatic changes in zebrafish fed different diets. Histopathological examination was performed on the same preparations as those used for visceral fat analysis and stained with Goldren trichrome. These sections were analyzed under a light microscope to assess the hepatic architecture, including the arrangement of hepatocytes around the sinusoids, and to identify any signs of steatosis, necrosis, or inflammation. This analysis aimed to detect histopathological changes indicative of liver damage due to dietary treatments.

### Statistical analyses

Data are presented as mean and standard deviation (SD). Differences between means of gross body measurements were tested using a main effects ANOVA, while feed analyses and visceral fat % were analyzed using a one-way ANOVA, both followed by a post hoc Tukey's honest significant difference test to correct for multiple comparisons. The statistical models presented below were used to analyze selected parameters:

$${\text{x}}_{{{\text{ij}}}} = \, \mu \, + \, \alpha_{{\text{i}}} + \, \beta_{{\text{j}}} + \, \varepsilon_{{{\text{ijk}}}}$$

and

$${\text{x}}_{{\text{i}}} = \, \mu \, + \, \alpha_{{\text{i}}} + \, \varepsilon_{{{\text{ik}}}} ,$$

where: x_ij_ or x_i_– the observation (body measurements parameters or feed parameters), i – the level of the first factor (group: NOD, HFD40, HFD60), j – the level of the second factor (time points, weeks: 12th and 20th), k – the number of measurements, µ – constant, general mean, α_i_ – main effect of the first factor, β_j_ – main effect of the second factor, ε_ijk_ – random error. There were no repeated measures as the data presented were derived from randomly selected animals. The normal distribution of the data was confirmed with the W Shapiro–Wilk test, and the equality of variance was verified with the Brown-Forsythe test. A two-sided significance level (P value) of less than 0.05 was considered statistically significant. GraphPad Prism version 10.0.2 for Windows (GraphPad Software, San Diego, CA, USA) was used for all statistical analyses.

### Institutional review board statement

The study was approved by the Local Ethics Committee for Animal Experiments (University of Life Sciences in Lublin, Poland, No. 100/2019). The methods were carried out in accordance with norms of the European Union law (Directive 2010/63/UE on the protection of animals used for scientific purposes, received in Poland by Legislative Decree 266/2015). The experiments were carried out at the Experimental Medicine Center of the Medical University of Lublin, Lublin, Poland in compliance with the ARRIVE guidelines.

### Editing software and language correction

As the authors of this manuscript are not native English speakers, MS Word Redactor, DipL Translate (https://www.deepl.com/translator) and DipL Write (https://www.deepl.com/write) were used to correct the English spelling, grammar, and style of the manuscript text.

## Results

**Figure 3:**
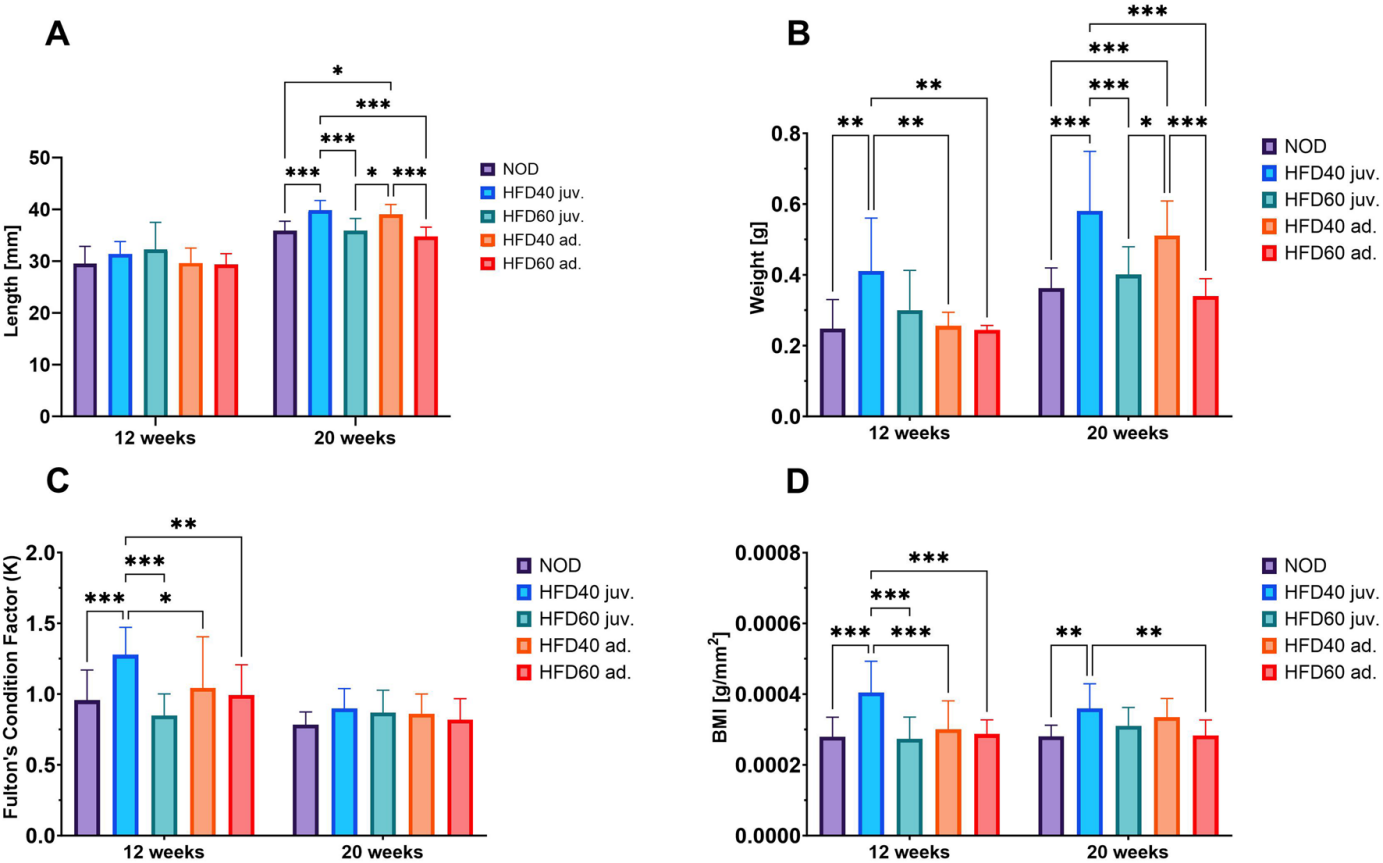
The effects of the innovative high-fat diet on zebrafish in 12 and 20 weeks of life: (**A**) Body weight (g), (**B**) Length (cm), (**C**) Fulton's Condition Factor K and (**D**) BMI (Body Mass Index). Bars represent means and whiskers represent SD; *P < 0.05; **P 0.01; ***P < 0.001. This figure summarizes the key parameters tracked in our study, showcasing the progression and impact of the diet on zebrafish obesity and health.

Fish that were given HFD40 beginning 2 weeks after fertilization, gained weight over 18 weeks due to fat accumulation in the abdominal cavity. Notably, the high-fat diet had a significant effect on the physiological traits of the fish. Furthermore, these fish had greater length and higher BMI than the control group (See Fig. 3). Similar results were observed in fish that were fed HFD40 from three months post-fertilization, with statistically significant outcomes. In contrast, comparable results were not observed in fish that received HFD60 for either 18 or 8 weeks (see Fig. 3). The experiments did not show any noticeable signs of dietary intolerance, symptoms of illness, or mortality.

Figure 3 illustrates results from two measurements taken at 12- and 20-week post-fertilization stages, which refer to the body length, weight and BMI of fish fed the HFD40 (juvenile and adult) and HFD60 (juvenile and adult) diets in comparison with the control group. Initial body length measurements were taken at 12 weeks post-fertilization, indicating no significant variance between the control and experimental groups. The final measurement was conducted at 20 weeks post-fertilization, upon completion of the experiment. Fish fed on HFD40 from the second week post-fertilization (juvenile) for 18 weeks demonstrated greater length in comparison to both the control group and those fed with HFD60 for the same period (P < 0.001). Comparable findings were noted in the fish that were administered HFD40 and HFD60 from the third month post-fertilization (adult). The HFD40-fed fish demonstrated an increase in length compared to the control group (P < 0.05). The study indicated that fish administered HFD40 during their juvenile period (i.e., from the second week post-fertilization) for a period of 18 weeks exhibited increased length in comparison to their adult counterparts that received HFD60 8 weeks after their third month post-fertilization. The difference was significant (P < 0.001) according to statistical analysis.

The weight measurement taken at the 12th week post-fertilization showed that the juvenile fish fed on HFD40 from the second week post-fertilization had gained more weight than both the control group and the group that received HFD60 during the same period (P < 0.01). Subsequently, the second measurement demonstrated significantly larger weight differences between HFD40 (juvenile) and the other groups (control, HFD60 juvenile, HFD40 adult and HFD60 adult). Fish fed HFD40 from the second week after fertilization for 18 weeks exhibited a significant increase in weight compared to all other groups (P < 0.001).

Regarding the BMI values obtained from the initial measurement, we noticed resemblances to the weight outcomes. After the second week post-fertilization (juv.), the fish that were given HFD40 had considerably increased BMI values in comparison to the control group and those that were fed with HFD60 for the same period (P < 0.001). During the second assessment, the juvenile fishes that were fed with HFD40 from the second week post-fertilization exhibited greater BMI values when compared to both the control group and the fishes fed with HFD60 from the third month post-fertilization (P < 0.01). However, no statistically significant differences were observed between the control group and the other groups.

The study observed differing fish conditions among experimental groups over the 12 weeks following fertilization, as indicated by the Fulton Condition Factor. The condition of the fish stabilized during the study, and no significant differences were observed at its conclusion. The results revealed that HFD40 had a greater impact on juvenile fish; this was reflected in a much higher weight/length ratio and a faster rate of fattening in this population. Fish fed with HFD40 exhibited markedly higher Fulton's Condition Factor than both the control and the juvenile group that was fed with HFD60 (P < 0.001 and P < 0.001, respectively). Interestingly, the juvenile group fed with HFD40 even exhibited a greater condition factor than both adult groups until 18 weeks post-fertilization (refer to Fig. 3).

**Figure 4:**
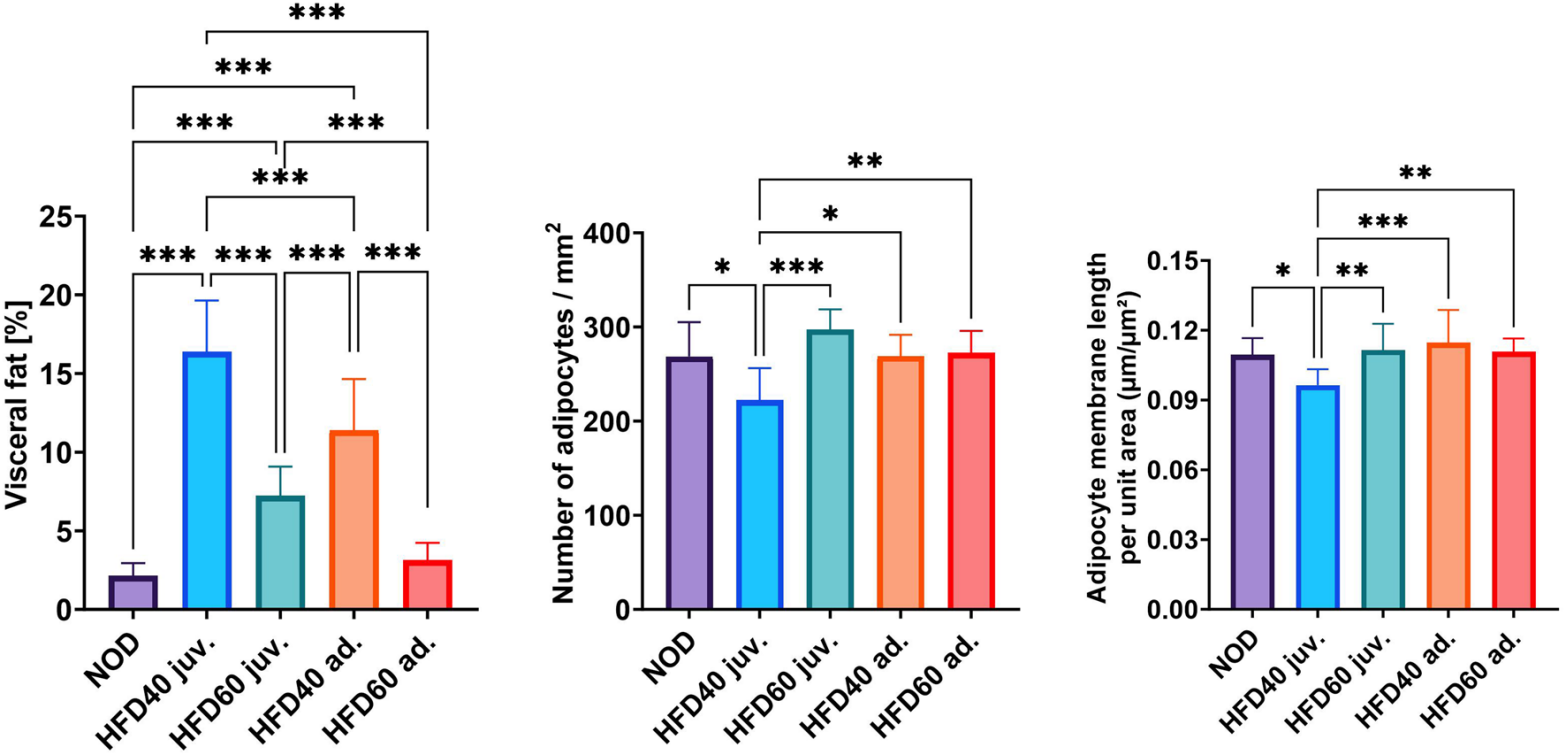
Impact of High-Fat Diet on visceral adiposity and adipocyte morphology in 20-week-old Zebrafish. This figure illustrates the effects of a novel high-fat diet on visceral fat content, adipocyte count, and adipocyte membrane complexity in 20-week-old zebrafish. The left panel quantifies the percentage of visceral fat in the abdominal cavity, the middle panel shows the number of adipocytes per square millimeter of tissue, and the right panel presents the adipocyte membrane length per unit area, indicating the changes in cell size and shape. Bars represent mean values and whiskers denote standard deviations. Statistical significance is denoted by asterisks: *P < 0.05, **P < 0.01, ***P < 0.001.

**Figure 5:**
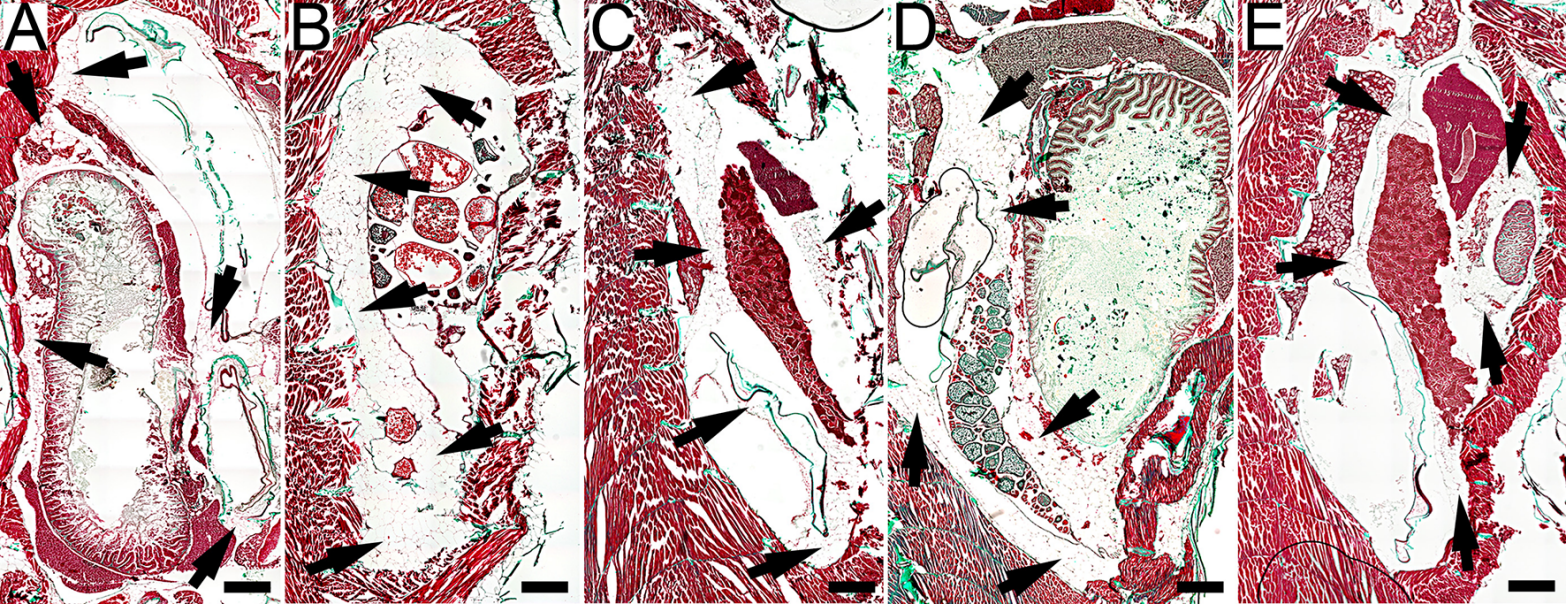
The impact of the innovative high-fat diet on the accumulation of visceral fat in the abdominal cavity of zebrafish at 12 and 20 weeks of age is shown. The spatial distribution of adipose tissue on the sagittal sections of zebrafish demonstrates the influence of the diet on zebrafish obesity. The diets were provided ad libitum using mechanical feeders—Eheim AutoFeeder (Deizisau, Germany). (**A**) The NOD control group received a standard commercial diet, GEMMA, supplemented with *Artemia salina* and containing 14% fat. (**B**) The HFD40 juv. group was fed a newly formulated diet starting from the second week of life after fertilization, which consisted of a commercial basal diet blended with 40% Biff Fat Powder and 10% magnesium aluminometasilicate, along with simultaneous provision of *Artemia salina*. (**C**) The HFD60 juv. group was fed a newly formulated diet starting from the second week of life after fertilization, which consisted of a commercial basal diet blended with 60% Biff Fat Powder and 10% magnesium aluminometasilicate, along with simultaneous provision of *Artemia salina*. (**D**) The HFD40 ad. group was fed a newly formulated diet starting from the 12th week after fertilization, which consisted of a commercial basal diet blended with 40% Biff Fat Powder and 10% magnesium aluminometasilicate, along with simultaneous provision of *Artemia salina*. (**E**) The HFD60 juv. group was fed a newly formulated diet starting from the 12th week of life after fertilization, which consisted of a commercial basal diet blended with 60% Biff Fat Powder and 10% magnesium aluminometasilicate, along with simultaneous provision of *Artemia salina*. Adipose tissue is indicated by arrows and scalebars are 500 µm long. Goldner's trichrome staining method was used to visualize histological preparations.

The proportion of visceral fat assessed at the end of the study is shown in Fig. 4. The spatial distribution of abdominal adipose tissue is shown in Fig. 5. These findings exhibit an evident connection between dietary consumption and the accretion of visceral fat in fish. In comparison to all other experimental groups, those in the control group exhibited the lowest percentage of visceral fat (P < 0.001). By contrast, fish that were given HFD40 starting in the second week after fertilization (juvenile stage) displayed a notably elevated proportion of visceral fat compared to all other groups, including the control group (P < 0.001). Similarly, fish that received HFD40 beginning in the third month after fertilization (adult stage) exhibited variances in visceral fat percentages compared to those that were given HFD60 for an equivalent period (P < 0.001). Fish fed HFD60 from the second week post-fertilization accumulated a greater amount of visceral fat in comparison to the fish that received the same diet from the third month post-fertilization (P < 0.001).

Quantitative analysis of the adipocyte morphology revealed significant variations among the dietary groups. As shown in Fig. 4 (middle and right graphs), the HFD40 juvenile group demonstrated the lowest number of adipocytes per square millimeter, indicating decreased adipocyte density compared with the other groups. Since there were no significant differences among adult groups and HFD60 juvenile group, also compared to the control, these results sug-gest a differential effect of fat concentration on adipocyte proliferation which was developmental dependent. In addition, measurement of adipocyte membrane length per unit area across groups revealed that HFD40 juvenile fish possessed the lowest proportion of adipocyte membrane length per unit area. This resembled the largest adipocytes because they had the highest visceral fat percentage (Fig. 4, left graph), which also potentially reflects increased lipid accumulation within individual cells. These morphological changes provide insights into microstructural alterations in visceral fat tissue in response to the high-fat diets.

**Figure 6:**
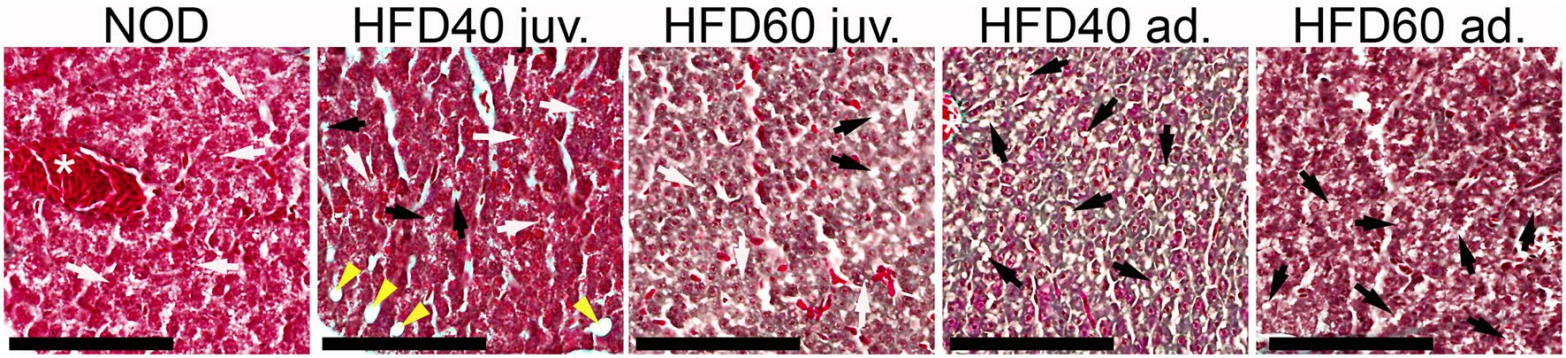
Histological evaluation of zebrafish liver tissue subjected to various dietary regimens. This figure illustrates the histopathological differences in the liver tissue among the zebrafish groups subjected to different dietary conditions, highlighting the impact of the high-fat diet on liver health. Liver sections were stained with Goldner's trichrome stain to examine cellular morphology. Control group (NOD): Zebrafish fed a standard commercial diet (GEMMA) exhibited typical liver architecture with varying degrees of minimal vacuolation. HFD40 juvenile group (HFD40 juv.) Zebrafish starting a high-fat diet from the second week post-fertilization were fed a diet blended with 40% Beef Fat Powder and 10% magnesium aluminometasilicate, and showed mild steatosis with mild vacuolation. HFD60 juvenile group (HFD60 juv.) Juvenile zebrafish, starting a high-fat diet from the second week post-fertilization, were fed a diet blended with 60% Beef Fat Powder and 10% magnesium aluminometasilicate, and displayed mild steatosis with moderate vacuolation. HFD40 adult group (HFD40 ad.) Adult zebrafish starting a high-fat diet from the 12th week post-fertilization were fed a diet blended with 40% Beef Fat Powder and 10% magnesium aluminometasilicate, demonstrating noticeable moderate steatosis but without large vacuolation. HFD60 adult group (HFD60 ad.) Adult zebrafish, starting a high-fat diet from the 12th week post-fertilization, were fed a diet blended with 60% Beef Fat Powder and 10% magnesium aluminometasilicate, showing mild to moderate steatosis with moderate vacuolation. All groups supplemented with *Artemia salina*. Key histological features, such as the 'muralium duplex' arrangement—yellow arrowheads, hepatocyte vacuolation—white arrows, and steatosis—black arrows, are indicated. Scale bars represent 100 µm. These images provide a comparative insight into the varying degrees of liver steatosis induced by different high-fat diets.

In the histopathological examination of zebrafish livers, preservation of the ‘muralium duplex’ structure was consistently noted across all experimental groups. This structure, comprising two layers of hepatocytes encircling sinusoidal blood vessels, is a defining characteristic of zebrafish liver histology. The present study found variations in cytoplasmic vacuolation of hepatocytes across different groups, which supports the recognized standards of zebrafish liver histology^33^. Interestingly, mild liver steatosis, as evidenced by the accumulation of fat in hepatocytes, was identified in groups subjected to high-fat diets, with noticeable variation depending on the duration of supplementation. The HFD40 ad. group exhibited a significant degree of steatosis, which did not extend to large vacuolation. Conversely, the HFD60 ad. group exhibited a less pronounced steatotic response. Crucially, no indications of advanced liver degeneration, including fibrosis, necrosis, parenchymal malformations, or inflammation, were detected in any group (Fig. 6).

These findings suggest that the high palmitic acid content in the beef fat-based diet induced mild steatosis in the zebrafish model yet did not precipitate severe liver damage under the study conditions. This emphasizes the unique influence of dietary fats on liver health in zebrafish, indicating a promising area for further investigation to discern the distinct consequences of different fatty acids.

The fatty acid profile of the diets differed significantly (Tables 1, 2, 3), depending on the composition of their ingredients. The SFA content can be represented as HFD60 > HFD40 > NOD. NOD contained more total fatty acids (including total PUFA, n-3 and n-6 PUFA) than the experimental diets and was also characterized by a higher PUFA/SFA ratio. The highest SFA content in the HFD60 diet was due to significantly higher levels of caprylic acid (C8:0), lauric acid (C12:0), palmitic acid (C16:0), stearic acid (C18:0) and arachidic acid (C20:0) compared to the NOD and HFD40 diets. The HDF40 diet contained the highest levels of behenic acid (C22:0) compared to the other diets. The higher content of total UFA in the NOD diet was due to the presence of palmitoleic acid (C16:1 n-7), heptadecenoic acid (C17:1) and especially the high content of docosahexaenoic acid (C22:6 n-3) and gondoic acid (C20:1 n-9), which were not found in the experimental diets. The highest amount of oleic acid (C18:1 n-9) was found in the HFD40 diet. CLA was most abundant in the NOD > HFD60 > HFD40 diet. The SFA/UFA ratio and the n-6/n-3 ratio can be expressed as HFD60 > HFD40 > NOD.

**Table 2:** Raw ash and raw fat concentration in commercial diet, HFD40 and HFD60.

	NOD	HFD40	HFD60
Raw ash	13.3 ± 0.02	13.8 ± 0.14	5.8 ± 0.01
Raw fat	18.7 ± 0.01	37.6 ± 0.30	46.8 ± 0.51

**Table 3:** Content of fatty acids in commercial diet, HFD40 and HFD60, fatty acid profile (g/100 g of total fatty acids) in zebrafish feed.

Fatty acids	NOD	HFD40	HFD60
Butyric 4:0	0.108	0.067	0.063
Caprylic 8:0			0.060
Lauric 12:0		0.280	0.302
Myristic 14:0	1.791	1.380	1.297
Pentadecanoic 15:0	0.229	0.102	0.069
Palmitic 16:0	18.29	43.03	48.45
Heptadecanoic 17:0	0.188	0.146	0.131
Stearic 18:0	3.795	4.832	5.196
Arachidic 20:0	0.337	0.400	0.448
Behenic 22:0		0.202	
Hexadecenoic 16:1 n9	1.924	0.413	0.192
Palmitoleic 16:1 n-7	0.122		
Heptadecenoic 17:1	0.211		
Oleic 18:1 n-9	12.55	35.35	34.33
Gondoic 20:1 n-9	1.600	0.375	0.234
Lineolic 18:2 n-6	33.50	11.80	8.590
Eicosadienoic 20:2 n-6	0.214	0.086	
Eicosatrienoic 20:3 n-3	3.456	0.544	0.264
Eicosapentaenoic 20:5 n-3	6.332	0.784	
Docosahexaenoic 22:6 n-3	13.46		
Conjugated linoleic CLA	0.670	0.214	0.370
Σ SFA	24.74	50.44	56.02
Σ MUFA	16.40	36.13	34.76
Σ PUFA	57.76	13.43	9.224
Σ UFA	74.16	49.56	43.98
Σ PUFA n-3	23.38	1.328	0.264
Σ PUFA n-6	33.71	11.88	8.590
Σ PUFA/SFA	2.335	0.266	0.165
Σ SFA/UFA	0.334	1.018	1.274
n-6/n-3	1.442	8.949	32.54

*SFA* saturated fatty acids, *MUFA* monounsaturated fatty acids, *PUFA* polyunsaturated fatty acids, *UFA* unsaturated fatty acids.

Zebrafish require 10 essential amino acids (Arg, His, Ile, Leu, Lys, Met, Phe, Thr, Trp and Val) for proper g rowth and development^19^. Table 4 displays the concentration of raw proteins in the diets under study, while Table 5 presents the amino acid profile of NOD, HFD40, and HFD60. Each diet contains 18 different amino acids, including the essential ones. The highest concentration was found in NOD. The lower concentration of amino acids in HFD40 and HFD60 didn't affect the growth of the zebrafish in the experimental groups.

**Table 4:** Raw proteins concentration in commercial diet (size of pellets 75,150,300) and composition with 80% concentration of beef fat.

Sample	Concentration (%)
Basal diet (size of pellets 75)	± 54.17
Basal diet (size of pellets 150)	± 55.84
Basal diet (size of pellets 300)	± 58.04
Biff fat powder	± 2.60

Biff Fat Powder (GRAUGmbH, Isselburg, Germany).

**Table 5:** Amino acids content in commercial diet, HFD40 and HFD60 [mg/g].

	NOD	HFD40	HFD60
Asp	45.3	24.8	14.4
Thr	21.3	11.5	6.69
Ser	21.4	11.0	6.38
Glu	80.1	41.8	24.2
Pro	58.0	28.1	14.8
Gly	32.9	17.4	9.71
Ala	29.8	15.6	8.90
Cysteic acid	8.84	4.66	2.65
Val	22.1	11.8	7.00
Sulf met	20.2	13.2	8.79
Ile	19.9	10.7	6.24
Leu	37.7	20.0	11.7
Tyr	17.5	9.27	5.55
Phe	21.4	11.1	6.58
His	13.0	6.94	3.64
Lys	33.7	17.9	10.2
Arg	30.9	16.0	8.78
Trp	5.14	2.09	1.69

## Discussion

In this study, we succeeded for the first time in creating a model of visceral obesity for fish feed for the entire juvenile term and separately for adults by overfeeding them with HFD40. Our study introduced a high-fat diet in zebrafish from the second week of life, continuing through their transition into adulthood. This approach allowed us to observe the accumulative effects of the diet across a critical developmental period, encompassing late juvenile stages through sexual maturity, as defined by our laboratory protocols and supported by the existing literature. Following a 10-week feeding period, the juvenile fish exhibited an increase in weight and BMI, which was further verified by calculating Fulton's Condition Factor. Continuing with constant overloading fish with lipids for another 8 weeks with HFD40, we observed even greater differences in the parameters. Furthermore, the fish in the experimental group were longer than those in the control group (refer to Fig. 3). Adult zebrafish that received an HFD40 diet for 8 weeks with the same feeding amount achieved the same outcomes (refer to Fig. 3). The group that was subjected to the HFD40 diet for 18 weeks had the highest percentage of visceral fat among the randomly chosen fish across all groups. Even the zebrafish exposed to the same diet for only 8 weeks managed to accumulate a significant amount of visceral fat compared to the control and other groups (see Fig. 4). Feeding fish a high-fat diet did not result in adipocyte hyperplasia in any of the experimental groups. Hypertrophic changes were observed in the group that received 40% beef fat during the juvenile stage. In the contrary in the same experimental group, we observed lower number of adipocytes compared to control and rest of experimental groups (see Fig. 4.)

In the histopathological examination of zebrafish livers of groups that received a high-fat diet from the third month post-fertilization, we detected mild steatosis, which is a symptom of nonalcoholic fatty liver disease (NAFLD). We did not observe the same changes in the livers of the groups that received a diet containing beef fat from the juvenile stage. These results show that depending on the stage of zebrafish development, the regeneration process in the liver and fat storage in hepatocytes are different. This model provides the possibility of examining changes in fat accumulation in the liver depending on the stage of zebrafish growth (see Fig. 6).

The zebrafish obesity model was developed using a newly prepared diet consisting of 40% beef fat and 14% carbohydrates, which was combined with typical zebrafish food (NOD) utilized for feeding in zebrafish facilities. NOD comprises approximately 50% proteins (as detailed in Tables 1 and 2), as well as 12% oil and other essential ingredients for the appropriate growth and development of zebrafish (as specified in Table 2). One of the fundamental components is ash, added to improve the food's stability in water conditions (refer to Tables 1 and 3). The New HFD mimics the Western diet to investigate if overfeeding with it creates parameters of visceral obesity. The study's results indicate that the appropriate concentration of fat to be added to NOD is 40%. By including 40% beef fat to the mixture, the proportions of the basal ingredients are altered with a decrease in concentration of proteins, oil, and ash. This HFD40 differs from the commonly used diet in creating zebrafish obesity models. Oka et al.^20^ published that overfeeding zebrafish with a high amount of *Artemia salina* (60 mg dry weight/day/fish) can result in the development of an obesity model. If excessive protein consumption occurs, such as with this dietary requirement, amino acids may be transformed into fat and deposited in adipose tissue^34^. This biochemical mechanism enabled the creation of an obesity model in which fish receive high amounts of *Artemia salina*. In zebrafish obesity models, standard diets often comprise high-fat components, such as dry egg yolk or various oils, supplemented with protein-rich *Artemia salina*. These diets are pivotal in inducing hyperlipidemia and weight gain, with *Artemia salina* specifically linked to hypertriglyceridemia and hepatosteatosis, underscoring the complexity of dietary influences on obesity phenotypes.

*Artemia salina* serves as a key source of protein for zebrafish, containing 40–60% crude protein (dry matter basis)^35^. Few studies have specifically evaluated the protein demands of zebrafish^36^. However, most available data suggests that a protein content of 30–53% in their food is sufficient^37^, although there is minimal information available on the quality of the protein^38,39^. HFD40 contains approximately 30% protein, and the fish were also given *Artemia salina* daily as an additional protein source. Presumably, the elevated level of dietary proteins was primarily utilized for elongating the body, rather than promoting the production of fat (see Fig. 3).

Other essential components for maintaining healthy zebrafish growth include oil, vitamins, and minerals, all of which are present in the NOD diet^40^ (Table 1). By incorporating fat into the NOD diet, the percentages of oil, fiber, and phosphorus decreased depending on the concentration of the added fat (Table 1). Of all the diets, HFD60 had the lowest proportions of these components. Despite changes in the diet composition, we did not observe any side effects or elevated mortality rates.

For the fat source, we utilized a mixture composed of 80% concentrated beef fat and 14% carbohydrate. Carbohydrate is not primarily essential; nevertheless, some researchers have reported that a deficiency of dietary carbohydrate results in reduced growth rates and impacts the transcriptome^41^. The studies indicate that excessive carbohydrate consumption is a leading cause of obesity in developed countries^42^. By adding carbohydrates to the HFD, we can increase the daily calorie intake.

Saturated fatty acids (SFA) and polyunsaturated fatty acids (PUFA) are among the key ingredients in every fabricated diet utilized for zebrafish feeding^43^. Fatty acids are renowned dietary components that offer energy and act as pheromones, hormones, and membrane constituents^44^. PUFA, including essential fatty acids such as linoleic (18:2 n-6) and linolenic acid (18:3 n-3), cannot be synthesized de novo by fish. Consequently, these substances must be obtained through dietary intake^45^. There is insufficient information regarding the minimum lipid requirements in relation to zebrafish. Meinelt et al. stated that growth, egg production and fertilization success require the presence of both n3 and n6 fatty acids^46,47^. Jaya-Ram et al. found that the fatty acid profiles of multiple organs changed when the source of dietary lipids was altered. They proposed that an appropriate balance of n3 to n6 lipid sources is crucial for achieving the best egg production and hatching rate^48^. Meinelt et al. reported that the ratio of n-3/n-6 PUFA in fish diet has a correlation with the enzyme activity responsible for elongating and denaturing fatty acids. The enzyme activity is especially related to water temperature. Cold water fish necessitate a higher intake of n-3 PUFA in their diet, whereas fish residing in warmer water require more n-6 PUFA. As zebrafish falls under the category of typical warmwater species, they require a higher n-6 PUFA intake in their diet^46^.

Saturated fats (SFAs) and polyunsaturated fats (PUFAs) were present in the factory-prepared diet used as the normal operating diet (NOD) in our experiment. The amount of monounsaturated fatty acids (MUFA) and unsaturated fatty acids (UFA) that make up the PUFA were measured, and the ratio of SFA/UFA was found to change depending on the percentage of added fat. As expected, since animal fat was used in creating the new high-fat diet (HFD), the concentration of SFA was higher in HFD60. The concentration of n-6 PUFA in HFD60 was unexpectedly high. Apparently, a higher concentration of added fat results in a higher percentage of n-6 PUFA within the diet. Considering the importance of these fatty acids in growth and fertilization, this finding holds particular significance.

During our experiment, we examined two High-Fat Diets (HFD40 and HFD60) that had varying fat concentrations. The findings demonstrate that overfeeding zebrafish with HFD40 leads to typical visceral obesity. Surprisingly, feeding the fish with HFD60 did not yield identical results. Notably, the results of morphometric measurements show that overfeeding zebrafish with HFD60 did not result in the typical obesity model. The fish were comparable in length, weight, and BMI to the control group (Fig. 3). The percentage of visceral fat, particularly in adult fish subjected to HFD60 for 8 weeks, was at a similar level to that of fish in the control group. Our findings suggest that the newly developed HFD60 may possess parameters like those of a ketogenic diet (KD).

The ketogenic diet (KD) is a specialized dietary regimen comprising low carbohydrate content, high fat concentration, appropriate protein, and other nutritive components. Technical terminology, such as "macronutrients," should be elucidated when first introduced. This diet primarily emphasizes high fat intake, moderate protein consumption, and low carbohydrate intake. Macronutrients are usually distributed in ranges of approximately 55% to 60% fat, 30% to 35% protein, and 5% to 10% carbohydrates^49^. The aim of the diet is to initiate ketosis, which is believed to modify metabolic pathways causing weight loss. Ketosis is believed to modify metabolic pathways, leading to weight loss and potential improvement of other health outcomes, including a reduction in hyperglycemia and enhancement of lipid profiles^50^. The ketogenic diet (KD) has demonstrated efficacy in promoting weight loss, diminishing hyperinsulinemia, and enhancing insulin sensitivity. Its effectiveness in such areas has been established through various studies^51^. The ketogenic diet has demonstrated benefits in treating drug-resistant epilepsy and other neurological disorders^52^.

The High-Fat Diet 60 (HFD60), which we formulated and administered to zebrafish, closely approximates a ketogenic diet. A formula containing high levels of fat was used to prepare this diet, which included carbohydrates. Based on the results of the tests and measurements of fish that were fed HFD60 for 18 and 8 weeks, it can be hypothesized that a novel ketogenic diet has been formulated that can be used for testing the effects of KD under laboratory conditions.

In the context of existing zebrafish obesity models, our study introduces a significant innovation in the development of an HFD40 diet. This high-fat diet, uniquely formulated with 40% beef fat and magnesium aluminometasilicate, sets our method apart from the traditional models that predominantly use dry egg yolk and various oils. Importantly, our diet effectively caused visceral obesity in zebrafish, a significant accomplishment that has not been previously achieved with other diets. This achievement underscores the potential of our model in closely mimicking the human condition of visceral obesity, thereby enhancing the relevance and applicability of our findings in obesity research.

In this paper we present the effects of overfeeding zebrafish with HFD40 (juvenile and adult) and HFD60 (juvenile and adult). Zebrafish fed HFD40 developed typical symptoms of visceral obesity. We have created a new model of visceral obesity in zebrafish. On the other hand, overfeeding with HFD60 resulted in no or insufficient weight gain and visceral fat percentage. This result was obtained with the specific parameters of HFD60, which in our opinion are close to KD. The mechanism is probably related to the development of the digestive system (for juveniles) and the absorption of specific fatty acids in the intestine. This needs further investigation.

## Patents

Newly prepared HFD40 is described in patent number Pat.242859.

## Data Availability

The data presented in this study are available on request from the corresponding author.
